# A clinical comparison of schizophrenia with and without pre-onset cannabis use disorder: a retrospective cohort study using categorical and dimensional approaches

**DOI:** 10.1186/s12991-015-0083-x

**Published:** 2015-12-10

**Authors:** Samuel Sarrazin, Florence Louppe, Raphael Doukhan, Franck Schürhoff

**Affiliations:** AP-HP, DHU PePSY, Hôpitaux universitaires Henri-Mondor, Pôle de Psychiatrie et d’Addictologie, 94000 Créteil, France; INSERM U955, Equipe 15, IMRB, 94000 Créteil, France; Faculté de Médecine, Université Paris-Est, 94000 Créteil, France; FondaMental Fondation, Fondation de coopération scientifique, 94000 Créteil, France

**Keywords:** Schizophrenia, Psychosis, Cannabis, Symptom dimension, OCCPI

## Abstract

**Background:**

A high prevalence of cannabis use disorder has been reported in subjects suffering from schizophrenia, fuelling intense debate about whether schizophrenia with pre-onset cannabis use disorder may be a distinct entity with specific features or whether cannabis use disorder can precipitate schizophrenia in genetically vulnerable subjects.

**Methods:**

We retrospectively assessed schizophrenia subjects with and without pre-onset cannabis use disorder on the basis of their clinical features, assessed categorically and dimensionally with the operational criteria checklist for psychotic illnesses (OCCPI). We also investigated whether the two groups could be differentiated on the basis of a history of psychiatric disorders in first-degree relatives. A principal component factor analysis of the OCCPI items was used to identify specific symptom dimensions. The relationships between symptom dimensions and cannabis status were analysed by point-biserial correlation analysis to control for sex and age at time of the assessment and illness duration.

**Results:**

One hundred and seventy-one subjects with a diagnosis of schizophrenia were included. Among them, forty-one patients (18.2 % of the sample) had a cannabis use disorder before or at the time of the onset of schizophrenia. We found similar results in symptoms patterns or family history between patients with and without pre-onset cannabis use disorder.

**Conclusions:**

Our results clearly argue against cannabis-associated schizophrenia being a relevant distinct clinical entity of schizophrenia with specific features.

## Background

A high prevalence of cannabis use has repeatedly been reported in psychotic patients [1]. Previous systematic reviews have reported a wide range (13–45 %) in the rates of cannabis use disorders (CUD) in schizophrenia users [2, 3], with a median lifetime rate estimated at 27.1 % [4]. Apart from the fact that acute intoxication can lead to ‘psychotic-like’ experiences that do not persist beyond the period of intoxication [5], various possibilities have been discussed for the association between cannabis and schizophrenia which include: (a) cannabis use is a component cause of schizophrenia; (b) the hypothesis of “self-medication” for symptoms of psychiatric diseases such as the negative symptoms of schizophrenia, anxiety, depression or dysphoria and (c) common socio-demographic factors and shared genetic factors.

Several studies have shown that the use of cannabis leads to physiological and cognitive deficits of a similar nature to those seen in schizophrenia [6]. In particular, there is an increasing body of evidence demonstrating that cannabis users (without schizophrenia) show deficits in tasks of sustained attention, working memory and other executive functions [7], as well as abnormalities in automatic processing of auditory stimuli: P50 suppression [8], prepulse inhibition (PPI) [9] and the mismatch negativity (MMN) [10] of the event-related potential (ERP). This suggests that dysfunctions in the endocannabinoid system could be involved in the development of similar deficits associated with cannabis use and schizophrenia, and further proposes that the neurobiology underpinning the development of electrophysiological/cognitive deficits in cannabis users may overlap with the neurobiological underpinnings of schizophrenia.

Other data suggest that the link between cannabis use and schizophrenia might be causal, even if a direct cause–effect relationship between the two disorders has not been clearly established. Systematic reviews regarding the association between cannabis and psychosis have suggested that cannabis can increase the lifetime risk of psychosis by a factor of two or three [11–13] and meta-analytic approaches found that the risk of psychosis was approximately 40 % higher in cannabis users than in non-users [14]. In the causal hypothesis, cannabis use may be directly involved in the pathophysiological processes that lead to a distinct form of schizophrenia-spectrum disorder that would have never happened otherwise. This is different from the hypothesis in which cannabis use may act as a precipitating factor in genetically vulnerable subjects. In this case, cannabis use would be expected only to precipitate the onset of schizophrenia. The causal hypothesis has been extensively discussed, and the diagnosis of “cannabis-induced schizophrenia” also known as “cannabis-associated schizophrenia” remains controversial [15]. Indeed, if “cannabis-associated schizophrenia” was a specific clinical entity, one would have expected to find specific clinical features including symptomatic profiles, course and prognosis.

Several studies have explored these hypotheses by comparing clinical characteristics between subjects who reported having used cannabis before the onset of schizophrenia and those who had not used cannabis before the onset of schizophrenia. Most studies have focused on samples of patients with a first-episode psychosis, making it possible to rule out the use of cannabis for self-medication [16] or to alleviate the side effects of antipsychotic medication. Several of these first-episode studies [17, 18] reported differences in clinical characteristics for individuals with cannabis use, including an earlier age at onset (for a meta-analysis, please refer to [19]). However, most of these studies found no such relationship [1, 20, 21], and failed to identify a specific clinical profile in cannabis users before the onset of the illness (for review, see [22]). These discrepancies might be due to uncertainties related to the diagnosis, including misclassifications of subjects during their first-psychotic episode. Similarly, studies exploring the symptom profiles of subjects with established schizophrenia with and without cannabis use have yielded conflicting results. Some have suggested that cannabis users had a different clinical profile, with more positive and less negative symptoms [23, 24], whereas others have found no difference between cannabis users or former users and non-users [25–27].

There is a relative lack of studies comparing specifically the clinical profiles of subjects suffering from schizophrenia with and without cannabis use before the onset of the illness. Such studies have again provided conflicting results concerning possible associations with various factors, such as premorbid social functioning, rates of positive and negative symptoms and overall prognosis [23, 26, 28–31]. Thus, it remains largely unknown whether cannabis-associated schizophrenia could be considered as a valid distinct clinical entity with a different course, including symptom expression and burden.

These discrepancies between studies may result from different factors. Firstly, the consequences of cannabis use on symptom profiles may be different in patients with CUD when compared to other cannabis users (e.g. through higher dose [32] or specific vulnerability). Secondly, discrepancies may result partly from differences in inclusion criteria or in the assessment procedures used. For example, in most studies, positive and negative symptoms are measured with clinical scales, such as the scale for the assessment of positive symptoms (SAPS), the Scale for the assessment of negative symptoms (SANS), the brief psychiatric rating scale (PBRS) and the positive and negative syndrome scale (PANSS), which are suitable for the assessment of symptoms in a cross-sectional approach, whereas the use of an instrument profiling symptoms over a lifetime perspective may possibly be more valid and reliable for assessments of the effects of cannabis use on schizophrenia. Of note, several studies analysing the factors underlying schizophrenia and other psychotic disorders have used the operational criteria checklist for psychotic illnesses (OCCPI) [33–36] and have shown that OCCPI factor analysis is a highly reliable method for lifetime dimensional phenotype description in schizophrenia.

To explore whether schizophrenia with pre-onset CUD could be a valid diagnostic entity with a specific symptomatic expression, we investigated the impact of pre-illness CUD on lifetime specific symptom patterns derived from the OCCPI in a relatively large sample of well-characterized subjects suffering from schizophrenia. We expected differences in symptom dimension profiles and clinical characteristics between schizophrenia subjects with and without pre-onset CUD. We also investigated whether it was possible to differentiate schizophrenia subjects with pre-onset CUD and those without on the basis of their history of psychiatric disorders in first-degree relatives. We hypothesized that subjects without pre-onset CUD would have a higher familial genetic liability to schizophrenia (higher rates of positive family history of schizophrenia) than schizophrenia subjects with pre-onset CUD. As far as we know, this is the first study to use OCCPI factor analysis to compare symptom profiles between schizophrenia subjects with and without pre-onset CUD in a lifetime perspective.

## Methods

### Subjects

Consecutively admitted subjects suffering from schizophrenia were recruited from a university-affiliated hospital (AP-HP, *Pôle de Psychiatrie et d’Addictologie*, Créteil, France). All probands included met DSM-IV-R criteria for schizophrenia and were interviewed by an experienced psychiatrist with the French version of the Diagnostic Interview for Genetic Studies (DIGS) [37] to confirm the diagnosis. Familial psychiatric morbidity was investigated with the Family Interview for Genetic Studies (FIGS) [38]. A complete family history for first-degree relatives was obtained from each subject and at least one first-degree relative. All subjects were euthymic at the time of the study, as evaluated with the Montgomery and Asberg Depression Rating Scale (MADRS) and the Beck-Rafaelson Mania Assessment Scale (MAS).

The study was reviewed and approved by the Research Ethics Board. All participants were provided with a complete description of the study and gave written informed consent.

### Clinical assessments

Socio-demographic characteristics, age at onset, number of hospital admissions and personal history of suicide attempts were recorded on the basis of both medical notes and data extracted from the DIGS. Patients were also assessed for lifetime cannabis and other substance use, and were asked to state precisely when use had begun, its duration and the mode of consumption. Patients were assigned to the “pre-onset CUD” group if they met lifetime DSM-IV-R criteria for cannabis use disorders (cannabis abuse or dependence) before or at the time of schizophrenia onset. Otherwise, they were assigned to the “no pre-onset” CUD group. Patients with other substance use disorders were excluded with an exception for tobacco smoking. Age at onset of schizophrenia was defined as the age at which the patient first met DSM-IV-R criteria for schizophrenia, according to medical case notes and interviews.

### Assessment of symptom dimensions and OCCPI ratings

The Operational Criteria Checklist for Psychotic Illness (OCCPI) [39] consists of 90 items assessing clinical characteristics and symptoms used in a wide range of operational diagnostic systems. We chose to use the OCCPI because (1) the presence of symptoms is recorded categorically; (2) a wide range of psychotic symptoms are recorded; (3) it is designed to assess symptoms that have occurred at some point in the subject’s life (lifetime perspective); (4) it records affective symptom data and (5) it can make use of data from different interviews. The OCCPI was completed, for the lifetime occurrence of symptoms by two raters using the DIGS and the information from interviews recorded in case notes. The inter-rater reliability (Kappa) of factorial scores was assessed for 30 test cases and was found to be very good [Kappa = 0.84, 95 % CI (0.59, 0.75)].

### Data analyses

#### Principal component analysis (PCA)

The aim of a principal component analysis is to reduce the number of variables used to describe a sample, by choosing linearly uncorrelated variables called principal components. All items defining a single symptom (*n* = 48) were chosen and coded dichotomously. We performed a principal component analysis on 28 symptoms of the OCCPI (20 symptoms were excluded due to a lack of variance or >10 % missing data). We extracted the initial factors and then performed an orthogonal rotation by the VARIMAX method. The number of meaningful factors was determined by the scree plot. For each of the symptom dimensions identified, OCCPI items with a loading greater than 0.4 were used to construct a quantitative scale. Subjects were scored by calculating the proportion of items present for each symptom dimension. Each subject was thus given a symptom pattern score for each of the scales.

The scree plot indicated that there were four substantive factors accounting for 46 % of the variance. The following symptom dimensions were identified: Factor 1 (affective: 11 items): loss of energy/tiredness, loss of pleasure, poor concentration, slowed activity, initial insomnia, poor appetite, dysphoria, excessive self-reproach, increased self-esteem, irritable mood and suicidal ideation; Factor 2 (reality distortion: 9 items): primary delusional perception, other primary delusion, delusions of passivity, bizarre delusions, auditory hallucinations (1), Clérambault-Kandinsky complex (2), non-affective hallucination of any type, delusions of influence and grandiose delusions; Factor 3 (disorganized/negative: 5 items): inappropriate affect, restricted/blunted affect, negative formal thought disorder, bizarre behaviour and positive formal thought disorder (3) and Factor 4 (motor: 3 items): delusions of guilt, agitated activity and catatonia. The affective factor accounted for 20.3 % of the total variance, reality distortion accounted for 12.4 %, the disorganized/negative factor for 7.0 % and the motor factor for 6.0 % (Table 1).

**Table 1:** Factor loadings for OCCPI items following varimax rotation

Principal component	Affective	Reality distortion	Disorganised/negative	Motor
Loss of energy/tiredness	*0.84*			
Loss of pleasure	*0.83*			
Poor concentration	*0.82*			
Slowed activity	*0.68*			
Initial insomnia	*0.63*			
Poor appetite	*0.62*			
Dysphoria	*0.6*			
Excessive self-reproach	*0.59*			
Increased self esteem	*0.48*			
Irritable mood	*0.47*			
Suicidal ideation	*0.4*			
Primary delusional perception		*0.76*		
Other primary delusions		*0.66*		
Delusions of passivity		*0.62*		
Bizarre delusions		*0.61*		
Auditory hallucinations^a^		*0.58*		
Clérambault–Kandinsky complex^b^		*0.57*		
Non-affective hallucination of any type		*0.54*		
Delusions of influence		*0.53*		
Grandiose delusions		*0.39*		
Inappropriate affect			*0.67*	
Restricted/ blunted affect			*0.66*	
Negative formal thought disorder			*0.56*	
Bizarre behaviour			*0.48*	
Positive formal thought disorder^c^			*0.44*	
Delusions of guilt				*0.56*
Agitated activity				*0.31*
Catatonia				*−0.62*

Highest factor loading for each item in italics type

Factor loading <0.3 are not shown

^a^Auditory hallucinations: third-person auditory hallucinations and/or running commentary voices and/or other (non affective) auditory hallucinations

^b^Clérambault–Kandinsky complex: thought insertion and/or thought withdrawal and/or thought broadcast and/or thought echo

^c^Positive formal thought disorder: positive formal thought disorder and/or speech difficult to understand and/or incoherent

#### Statistical methods

Data were analysed with PASW Statistics version 18.0 (IBM, Chicago, Ill, USA). We compared schizophrenia subjects with and without pre-onset CUD in terms of demographic and clinical variables, including sex, family history of psychiatric disorders, age at onset of schizophrenia, illness duration, personal or familial history of suicide attempts. The differences between groups were assessed with the Student *t* test or Man-Whitney test for continuous variables and Pearson’s Chi-squared or Fisher’s exact tests for discrete variables, and a *p* value <0.004 adjusted for Bonferroni correction was considered statistically significant. The relationships between symptom dimensions and CUD status were analysed by Spearman point-biserial correlations to control for the potential confounding influences of sex, age at the time of the assessment and illness duration.

We also carried out logistic regressions to assess the effects of categorical and dimensional variables on the likelihood that patients had CUD before the onset of schizophrenia. We included in the first model only categorical variables that were found significantly different between the pre-onset CUD and “no pre-onset” CUD schizophrenia groups (sex, age at assessment and duration of illness). The second model included both categorical and dimensional variables. Duration of illness was removed from the models because of a risk of singularity between “age at assessment” and “duration of illness”. All assumptions of logistic regression models were met, including independence of cases, exclusion of multicollinearity and linear relationship between continuous independent variables and the logit transformation of the dependent variable. We estimated the variance explained by predicting variables using Nagelkerke’s R^2^.

## Results

### Sample characteristics

The initial sample consisted of 207 subjects diagnosed with schizophrenia. Thirty-six subjects (17.4 %) had a DSM-IV-R lifetime diagnosis for a substance use disorder other than cannabis and were thus excluded.

The final sample was composed of 171 subjects and was predominantly males (67.1 %). The mean age at assessment was 34.0 years (SD 11.7). The mean age at onset was 23.7 years (SD 7.9), and the mean duration of illness was 11.5 years (SD 11.1).

### Pre-onset CUD vs. “no pre-onset” CUD: categorical variables

Demographic and clinical characteristics are detailed in (Table 1). Thirty-five subjects (20.5 % of the total sample) met DSM-IV-R criteria for CUD (cannabis abuse or dependence) without comorbid other substance use disorder. Among the 35 subjects with CUD, 31 subjects began using cannabis before or at the time of schizophrenia onset and were thus assigned to the pre-onset CUD group. There were significantly fewer women in the pre-onset CUD group and these subjects were younger than those in the “no pre-onset” CUD group.

The mean age at onset of schizophrenia did not differ significantly between the two subgroups following Bonferroni correction. The pre-onset CUD group had a shorter duration of illness than the “no pre-onset” CUD group. There was no difference in the number of hospital admissions per year between the two groups. There were also no significant differences between the two groups in terms of the proportions of people with a positive family history of schizophrenia, mood disorders or suicide attempts (Table 2).

**Table 2:** Comparisons of the demographic and clinical characteristics of subjects belonging to the schizophrenia (SZ) with and without pre-onset cannabis use disorder (pre-onset CUD)

	Total sample	SZ with no pre-onset CUD	SZ with pre-onset CUD	*p*
Number of subjects	171	140	31	–
Male [n (%)]	114 (67.1%)	*84 (60.4%)*	*30 (96.8%)*	*<0.001*
Mean age at assessment [years (SD)]	34.0 (11.7)	*35.4 (12.1)*	*27.6 (6.8)*	*0.001*
Mean age at onset [years (SD)]	23.7 (7.9)	24.4 (8.5)	20.9 (3.4)	0.027
Mean age at cannabis use onset [years (SD)]	–	–	18.3 (3.7)	–
Mean duration of the illness [years (SD)]	11.5 (11.1)	12.6 (11.7)	6.7 (6.7)	0.007
Mean number of hospital admissions/illness duration	0.6 (0.7)	0.6 (0.8)	0.6 (0.4)	0.82
Personal history of suicide attempts [n (%)]	75 (45.5%)	65 (47.8%)	10 (34.5%)	0.19
Family history of schizophrenia [n (%)]	16 (10.2%)	12 (9.4%)	4 (13.3%)	0.53
Family history of mood disorders [n (%)]	66 (44.0%)	56 (46.3%)	10 (34.5%)	0.25
Family history of suicide attempts [n (%)]	26 (17.6%)	23 (19.3%)	3 (10.3%)	0.25

Significant p values after Bonferroni correction (threshold p < 0.004) are presented in italics

*SD* standard deviation, *n* number

### Pre-onset CUD vs. “no pre-onset” CUD: symptom dimension features

**Fig. 1:**
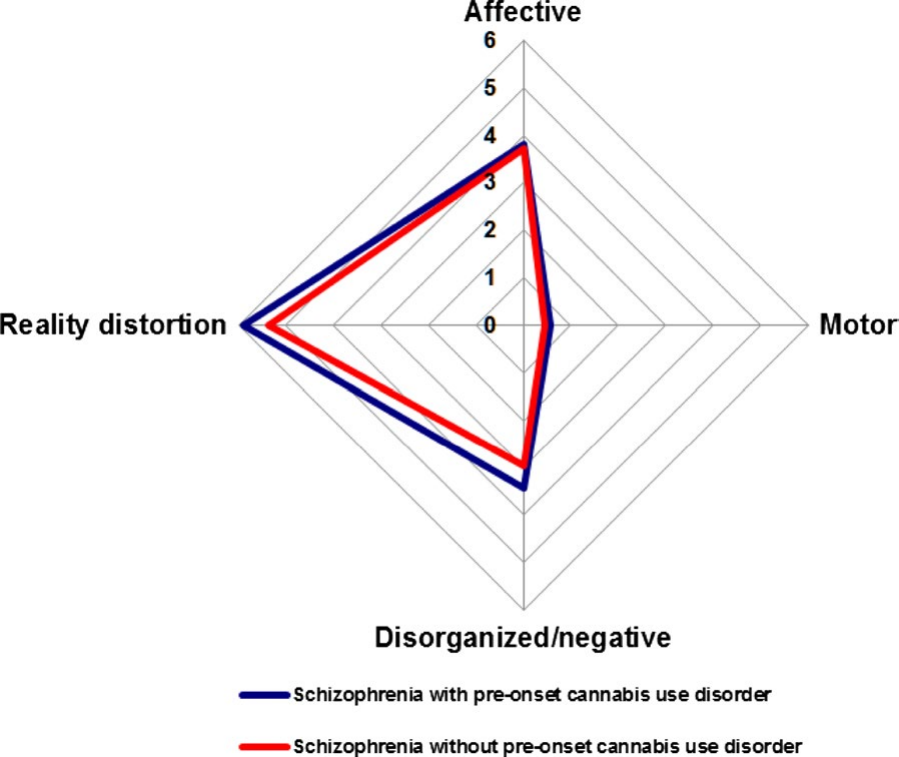
Comparisons of symptom dimensions (factor scores) between schizophrenia with and without pre-onset cannabis use disorder

No significant difference between the groups was found for any of the symptom dimensions (affective (*r* = 0.05; *p* = *0.46*), reality distortion (*r* = 0.08; *p* = *0.34*), disorganized/negative (*r* = 0.04; *p* = *0.62*), motor (*r* = 0.04; *p* = *0.63*)) after controlling for sex and age at the time of the assessment) (Fig. 1).

### Logistic regressions

The first logistic regression model was statistically significant [*X*^2^(2) = 28.47, *p* < 0.001]. The proportion of variance explained was 25.1 %. On the two variables included in this first model, male and younger subjects had higher likelihood of belonging to the pre-onset CUD group with respective odds of 15.76, IC95 = (2.06–120.43) and of 0.94, IC95 = (0.89–0.98).

The second logistic regression model included both demographical variables and the four factor scores. The results were not substantially changed. The model remained significant [*X*^2^(6) = 30.32, *p* < 0.001] and explained 26.6 % of the variance group difference. Male and younger patients were more likely to be classified to the schizophrenia with pre-onset CUD group, while no factor score was significantly associated with group membership when controlling for demographic variables (Tables 3, 4).

**Table 3:** Logistic regression predicting likelihood of schizophrenia with pre-onset cannabis use disorder for the first model including categorical variables

	*B*	SE	*df*	*p*	Odds ratio	95% Confidence interval for odds ratio	
						Lower	Upper
Sex^a^	2.76	1.04	1	*0.008*	*15.76*	2.06	120.43
Age at assessment	−0.06	0.02	1	0.007	0.94	0.89	0.98

^a^Male gender is coded “1” and female gender “0”, patients with pre-onset CUD were coded “1” and those without “0”

**Table 4:** Logistic regression predicting likelihood of schizophrenia with pre-onset cannabis use disorder for the second model including categorical variables and factor scores

	*B*	SE	*df*	*p*	Odds ratio	95% Confidence interval for odds ratio	
						Lower	Upper
Categorical variables							
Sex^a^	2.79	1.05	1	*0.008*	*16.29*	2.09	126.75
Age at assessment	−0.07	0.03	1	0.008	0.93	0.89	0.98
Factor scores							
Affective	0.06	0.08	1	0.47	1.06	0.91	1.14
Reality distortion	0.09	0.09	1	0.31	1.10	0.92	1.32
Disorganised/negative	0.02	0.17	1	0.91	1.02	0.72	1.43
Motor	0.07	0.24	1	0.77	1.07	0.66	1.73

^a^Male gender is coded “1” and female gender “0”, patients with pre-onset CUD were coded “1” and those without “0”

## Discussion

We carried out a detailed comparison of schizophrenia subjects with and without pre-onset CUD, using categorical and lifetime dimensional approaches. Contrary to our expectations, we did not find any clear symptom pattern differentiating the two groups of subjects.

### Categorical approach

The lifetime prevalence rate of CUD in our sample is within the range of lifetime prevalence rates (13–45 %) previously reported in schizophrenia [1, 3] and is very similar to the rate of 27 % reported by Koskinen et al. [4]. Men and women account for similar proportions of the schizophrenia population, but most controlled clinical trials have found men to be over-represented among substance users with schizophrenia [24, 40–44]. The proportion of men was higher in our pre-onset CUD group than in the “no pre-onset” CUD group, which included only four patients with CUD beginning after the onset of schizophrenia and mostly patients without lifetime CUD. However, this predominance of men among cannabis users is not specific to psychotic disorders. In a recent survey, it was estimated that rates of cannabis use during the past month were about 10 % for male subjects and 6 % for female subjects [45].

Several studies [44, 46–48] but not all [49–51] reported that substance users have an earlier onset of the disease. More recently, a meta-analysis has confirmed the link between cannabis use and an earlier age at onset of schizophrenia [19]. However, the studies included in this meta-analysis did not take into account whether the substance was used prior to the onset of psychosis, or later in the course of established schizophrenia. In our sample, the age at onset was not different between the two groups of patients, thus not favouring the hypothesis of the illness being triggered by substance use as it has been suggested in people predisposed to psychosis [12]. Subjects with schizophrenia and substance use have been reported to have a history of more lifetime suicide attempts [52, 53]. In our sample, the frequency of suicide attempts was not different between subjects with and without pre-onset CUD. Thus, our results with others [54] do not favour the hypothesis that CUD prior the onset of schizophrenia acts as a mediator of suicidality.

In accordance with some [24, 42] but not all previous studies [55], we found no difference between the groups in terms of the percentage of patients with a positive family history of schizophrenia using a careful semi-standardized familial assessment. This result also adds weight to the criticisms of the validity of the diagnosis of “cannabis-associated schizophrenia” being a distinct clinical entity. Indeed, if hereditary predisposition had been found to differ between subjects with pre-onset CUD and “no pre-onset” CUD, this might have provided some indirect support for the validity of this diagnosis.

### Dimensional approach

We found no difference in lifetime symptom dimensions between subjects with schizophrenia according to their personal history of CUD before the onset of schizophrenia. The magnitudes of the various symptom dimensions were very similar in the two groups. Most studies comparing cannabis users and non-users at the time of a first-psychotic episode or in a context of chronic schizophrenia did not evidence a relationship between cannabis use and symptoms [4, 20, 24, 42, 56–58]. A few studies have reported evidence of association with greater positive symptoms [59–61] or lesser negative symptoms [23, 62, 63]. Methodological differences may explain such discrepancies. Indeed, using a reliable lifetime dimensional approach, our findings clearly argue against “cannabis-associated schizophrenia” being a distinct clinical entity.

### Strengths and limitations of the study

The major advantage of this study is the use of a factorial analytical method to identify lifetime symptom dimensions related to a specific psychopathological domain. Our sample consisted of well-characterized subjects, and the information about family history was also carefully collected with a semi-standardized instrument. We also chose to include subjects with established schizophrenia rather than first-episode subjects to reduce the risk of uncertainties related to the diagnosis. Finally, we excluded subjects with other substance abuse or dependence to avoid biases related to other substance such as alcohol or opiates.

Several methodological limitations should be considered. Firstly, we defined our group based on their history of abuse or dependence to cannabis before the onset of schizophrenia and not their consumption. The rationale for our decision was driven by retrospective design. Indeed, retrospective self-report of cannabis use (e.g. by reporting daily dose) might be highly impacted by recall and declaration biases. To improve data accuracy, we used a structured assessment tool that has a very good reliability to assess CUD [64]. The second limitation is that we cannot rule out an effect of medication use on the clinical profiles of subjects. Thirdly, our dimensional approach did rely on a principal component analysis that did not distinguish separate negative and disorganized dimensions in our sample. We therefore cannot exclude the possibility of an association between pre-onset CUD and one of these dimensions. Finally, in our PCA, the extracted components explained 46 % of the variance in the symptom data being recorded. This may seem low, but this value is in the range seen in all the studies using the same instrument and the same statistical methodology (mean: 52.2 % range 39–71 %).

## Conclusions

Finding differences between schizophrenia subjects with and without pre-onset CUD in terms of symptom patterns would be of interest. Indeed, this would suggest that the pathological mechanisms underlying the symptoms of schizophrenia when associated with a pre-onset CUD can be induced by a direct pharmacological effect of cannabis use. This would have provided some validity to a putative “cannabis-associated schizophrenia” diagnosis. Taken together, our results clearly do not support the hypothesis of “cannabis-associated schizophrenia” being a distinct nosographic entity. Firstly, we found no specific symptom profiles in the pre-onset CUD group. Secondly, we found that gender and a younger age were associated with the pre-onset CUD group. However, these factors have been previously identified in samples of cannabis users without psychosis [59, 60] and are non-specific factors. Thirdly, we did not find a higher percentage of positive family history of schizophrenia in the “no pre-onset” CUD group, thus not supporting the hypothesis that genetic factors contributes more significantly in this group. Future prospective birth cohort and population studies evaluating neuroimaging, neuropsychological, negative life events and genetic parameters with larger sample sizes remain needed to better understand the link between cannabis use and schizophrenia.
